# Empathy levels among health professional students at a large midwestern public university - a cross-sectional study

**DOI:** 10.1186/s12909-023-04090-x

**Published:** 2023-02-20

**Authors:** Kelsey Wenger, Lauren Reist, Andrea Achenbach, Kimberly Dukes, Michelle Fravel, Laura Knockel, Francis Kuehnle, Jeffrey Reist, Manish Suneja, Chandler Pendleton, Xian Jin Xie, Leonardo Marchini

**Affiliations:** 1 The University of Iowa College of Dentistry and Dental Clinics, Iowa City, IA USA; 2 The University of Iowa College of Pharmacy, Iowa City, IA USA; 3 The University of Iowa College of Nursing, Iowa City, IA USA; 4 The University of Iowa Roy J. and Lucille A. Carver College of Medicine, Iowa City, IA USA; 5 Case Western Reserve University School of Dental Medicine, Cleveland, OH USA; 6 Department of Comprehensive Care, Case Western Reserve University School of Dental Medicine, 9601 Chester Ave, 44106 Cleveland, OH USA

**Keywords:** Attitude of Health Personnel, Compassionate care, Dental Education, Empathy, Health Personnel, Medical Education, Pharmacy Education, Nursing education

## Abstract

**Background:**

Empathic care is considered extremely important by patients and providers alike but there is still an ample need for assessing empathy among healthcare students and professionals and identifying appropriate educational interventions to improve it. This study aims to assess empathy levels and associated factors among students at different healthcare colleges at the University of Iowa.

**Methods:**

An online survey was delivered to healthcare students, including nursing, pharmacy, dental, and medical colleges (IRB ID #202,003,636). The cross-sectional survey included background questions, probing questions, college-specific questions, and the Jefferson Scale of Empathy-Health Professionals Student version (JSPE-HPS). To examine bivariate associations, Kruskal Wallis and Wilcoxon rank sum tests were used. A linear model with no transformation was used in the multivariable analysis.

**Results:**

Three hundred students responded to the survey. Overall JSPE-HPS score was 116 (± 11.7), consistent with other healthcare professional samples. There was no significant difference in JSPE-HPS score among the different colleges (P = 0.532).

**Conclusion:**

Controlling for other variables in the linear model, healthcare students’ view of their faculty’s empathy toward patients and students’ self-reported empathy levels were significantly associated with students’ JSPE-HPS scores.

## Introduction

Compassion is defined as the emotional response to another person’s pain or suffering and it involves an authentic desire to help.[1] It differs from empathy, which is related to the feeling and understanding another person’s pain or suffering, because compassion involves taking action. Functional magnetic resonance studies showed that empathy activates the pain centers in the brain,[2] and compassion activates the reward pathway associated with affiliation and positive emotions.[3] However, due to their interconnection, empathy and compassion are often used interchangeably in the healthcare literature.

Empathic care is considered extremely important by patients and providers alike, and there is abundant evidence that providing empathic and compassionate care improves healthcare outcomes.[4] An empathic healthcare provider can encourage and motivate patients to take part in their treatment,[5–7] resulting in better outcomes,[6, 8–10] better patient communication,[11, 12] reductions in recovery time,[8] a decrease in reported pain,[8, 13] a decrease in fear of healthcare,[12, 14] an increase in patient satisfaction,[8] and a decrease in malpractice litigation.[15, 16] Providing compassionate care can also improve provider well-being[17] and the enhance the performance of the health care system.[18].

However, evidence is also mounting that there is a empathy crisis among healthcare providers in the United States healthcare system.[4] Frequently, providers miss opportunities to acknowledge patients’ feelings during routine office visits,[11] and fail to elicit patients’ concerns and listen attentively to them.[19] In addition, it seems that empathy levels decline over time during coursework in healthcare schools[20–23] suggesting a need to devote more attention to the development of compassionate care skills during healthcare education.[24] A strategy encompassing a framework for providing consistent feedback on healthcare students’ ability to display empathy could facilitate the development of more empathetic clinicians. [24]

Empathy decline during healthcare professional coursework has previously been found to be associated with numerous factors, including inappropriate role models, high workload, students’ personality and biography, hidden curriculum (implicit expectations), number of hours worked per week, and the hours slept per night.[14, 20, 25–27] Although interventions are effective in improving empathy levels among healthcare students,[28–30] widespread adoption of such assessments and interventions are needed.[31].

Studying the changes in empathy using a widely validated scale throughout healthcare professional curriculum may result in better understanding of how empathy levels change during student education and provide guidance for improved learning strategies aimed at developing a more empathetic healthcare workforce in the future. The aim of this study is to assess empathy levels and predictors of empathy among students at different healthcare colleges at the University of Iowa.

## Methods

This was a cross-sectional survey study among healthcare colleges at the University of Iowa, including nursing, pharmacy, dental, and medical students (all classes and programs offered). Each student was invited through email to participate in the study. The invitation also explained the purpose of the study and assured participants about confidentiality. Students received the invitation three times throughout the course of the spring semester of 2021 (Jan-June). The study was approved by the University of Iowa Institutional Review Board (IRB ID #202,003,636). All steps of the experiment were performed in accordance with relevant guidelines and regulations, and informed consent was obtained from all subjects and/or their legal guardian(s).

Demographic data was collected, including ethnicity, sex assigned at birth, gender, and mother’s maiden name (for future pairing). A section of probing questions related to factors previously associated with empathy levels [14, 20] was included, using a 5-point Likert scale. Furthermore, each college added additional questions for their specific college’s participants such as program type or desire to continue onto a residency program.

In addition, all students received the Jefferson Scale of Physician Empathy- Health Professions Student version (JSPE-HPS). The JSPE-HPS is a 20-item, psychometrically validated scale widely accepted to measure empathy among healthcare professionals. The median item-total score correlation of the JSPE-HPS has been reported to be statistically significant (0.42), and the internal consistency of the scale, as measured by the Cronbach’s coefficient α, was 0.78, which falls into the accepted standard. Test-retest reliability coefficients were also acceptable at 0.58 (within 3 months interval) and 0.69 (within 6 months interval) between testing.[32].

The Jefferson Scale of Empathy defines empathy as a predominantly cognitive (rather than an emotional) attribute that involves an understanding (rather than feeling) of experiences, concerns, and perspectives of the patient, combined with a capacity to communicate this understanding. The JSPE-HPS contains 20 7-point Likert scale items, ranging from 1 (strongly disagree) to 7 (strongly agree). Therefore, participants were scored 20–140, with a higher score indicating a higher empathy level. [33]

There are two versions of the Jefferson Scale of Empathy for students. One was designed for medical students (S-version) and the other is most appropriate for other health professions (HPS-version). All scales are similar in content with minor modifications in wording to fine tune the instrument to its target audience. [33]

### Statistical analysis

Data was collected using RedCap. To examine bivariate associations, Kruskal Wallis tests were used when three or more groups were being compared. When only two groups were being compared, Wilcoxon rank sum tests were used. Pairwise comparisons were also run on any significant Kruskal Wallis results to see which, if any, groups have empathy scores that were statistically significantly different. Pairwise p-values were adjusted using the Benjamini & Hochberg method. No adjustments were made to the Kruskal Wallis or Wilcoxon p-values to account for the multiple tests that were run.

The bivariate results were used to determine variables of interest for the starting multivariable model. Any variable with a p-value < 0.2 in the bivariate analysis was considered in the starting model, but gender was forced in regardless of p-value. Respondents’ view of faculty empathy toward the respondent, faculty empathy toward patients, impact of patients’ attitudes towards the respondent on the respondent’s empathy towards the patient, respondents’ own empathy, respondents’ stress levels throughout the week, and gender were all considered in the starting multivariable model, which was constructed to evaluate the associations of the covariates of interest and the JSE score.

After trying a few transformations of the outcome variable including a log transformation, a Boxcox transformation, and a square root transformation, it was decided to move forward using a linear model with no transformation since that model produced an adequate fit of the full model and led to more practical, relevant interpretations of the coefficients. Although the final model does show some slight non-normality, there does not seem to be much deviation when looking at the QQ plot. After deciding which model to use, backwards variable selection using AIC was utilized to determine the final model.

## Results

Three-hundred students responded to the survey (14.4% response rate). Table 1 presents demographics and mean JSPE-HPS scores of respondents by college. The majority of respondents were Caucasian females reporting their gender as women. The overall mean JSPE-HPS score was 116 (± 11.7), and there was no statistically significant difference in JSPE-HPS scores between colleges (P = 0.532).

**Table 1:** Respondents’ ethnicity, sex assigned at birth, gender, and JSPE-HPS average score by college

		All	College of Dentistry	College of Medicine	College of Nursing	College of Pharmacy
**Ethnicity**	**Caucasian**	238 (79.3%)	41 (67.2%)	33 (80.5%)	87 (86.1%)	77 (79.4%)
	**Other**	62 (20.7%)	20 (32.8%)	8 (19.5%)	14 (13.9%)	20 (20.6%
**Sex**	**Female**	235 (78.3%)	38 (62.3%)	30 (73.2%)	96 (95.0%)	71 (73.2%)
	**Male**	65 (21.7%)	23 (37.7%)	11 (26.8%)	5 (4.95%)	26 (26.8%)
**Gender**	**Man**	67 (22.3%)	24 (39.3%)	12 (29.3%)	5 (4.95%)	26 (26.8%)
	**Non-Binary**	1 (0.33%)	0 (0.00%)	0 (0.00%)	1 (0.99%)	0 (0.00%)
	**Woman**	232 (77.3%)	37 (60.7%)	29 (70.7%)	95 (94.1%)	71 (73.2%)
**JSPE-HPS Score**		116 (11.7)	115 (15.6)	116 (10.8)	118 (9.50)	116 (11.2)

In the College of Medicine, there was not a statistically significant difference in JSPE-HPS scores between the students’ graduation class. In the College of Dentistry, there was a statistically significant difference in JSPE-HPS score among the dental classes (p < 0.001). These differences seem to be driven by the D3 (third year dental student) class. This class has the lowest median JSPE-HPS score. After analyzing the pairwise comparisons and adjusting the p-value, there were no statistically significant pairwise differences between the types of practice that dental students are planning on going into.

In the College of Nursing, there was not a statistically significant difference in JSPE-HPS scores among the students in the 3-year BSN to DNP program, 4 year BSN to DNP program, Post MSN-DNP program, or MSN-CNL programs. There was not a statistically significant difference among students with previous RN experience (+/- 5years) nor between the nursing students who were admitted directly from high school vs. standard admission.

In the College of Pharmacy, there was a statistically significant difference among students pursing pharmacy residencies. Those who do not plan to pursue a pharmacy residency had a lower median JSPE-HPS score compared to those who planned to pursue a residency and those who were unsure about a residency (p = 0.005). There was also a statistically significant difference among those in dual degree programs. Students who are not completing any dual pharmacy/other certificate/program degree had a lower median JSPE-HPS score (p = 0.038). There was not a statistically significant difference in JSPE-HPS among the pharmacy classes, although P3 year had the highest median JSPE-HPS score (120). There was not a statistically significant difference between students based upon work or research experiences which included those with experience in community pharmacy, hospital inpatient pharmacy, hospital outpatient pharmacy, long term care pharmacy, research pharmacy, other pharmacy experience, or no pharmacy experience. There was also no statistically significant difference in JSPE-HPS scores between prior degrees obtained.

Table 2 presents the bivariate analysis among JSPE-HPS and the remaining covariates. Significant differences were found for students’ perceptions of faculty empathy towards both students and patients, and for respondents’ self-assessed empathy levels.

**Table 2:** Bivariate analysis among JSPE-HPS and covariates

Covariate		P-value / Median JSPE-HPS score
**Race/Ethnicity**		0.514
	*Caucasian*	118
	*Other Race/Ethnicity*	120
**Gender**		0.593
	*Man*	119
	*Woman*	118
**Sex**		0.800
	*Male*	119
	*Female*	118
**To what extent do you feel your faculty is empathetic to you?**		< 0.001*
	*To a very great extent*	120
	*To great extent*	119
	*To some extent*	116
	*To a little extent/Not at all*	114
**To what extent do you feel your faculty is empathetic to THE PATIENTS?**		< 0.001*
	*To a very great extent*	122
	*To great extent*	118
	*To some extent*	115
	*To a little extent*	113
**How much does your empathy level towards a patient depend on the patient’s attitudes toward you?**		0.105
	*To a very great extent*	121
	*To great extent*	116
	*To some extent*	118
	*To a little extent*	118
	*Not at all*	122
**How would you rate your OWN empathy?**		< 0.001*
	*Not very empathetic*	*113*
	*Very empathetic*	120
**During your coursework in professional school, did you have classes DEDICATED to empathy or compassionate care?**		0.740
	* A lot of classes*	*121*
	*Some classes*	118
	*A few classes*	118
	*Not at all*	118
**During a typical week, how would you describe your stress level?**		0.181
	*Very high stress*	*116*
	*Some stress*	119
	*Medium level or very low stress*	118
**During a typical week, how many times do YOU feel hurried when seeing patients?**		0.672
	*All the time*	*118*
	*Many times*	118
	*Sometimes*	119
	*A few times/never*	119
	*Not applicable*	118
**Outside of class and classwork, how many hours a week do you work?**		0.661
	*None (0 h)*	*118*
	*1–10 h*	118
	*11–20 h*	118
	*More than 20 h*	118

A * denotes statistically significant difference. Wilcoxon rank-sum tests were used to analyze variables with two groups; Kruskal Wallis tests were used to analyze variables with three or more groups.

Table 3 presents the final model for the multivariable analysis. In the final model, respondents’ view of their faculty’s empathy toward patients and respondents’ self-reported empathy levels were statistically significantly associated with the JSPE-HPS scores. Feeling that faculty is empathetic toward the patients to “some extent” was associated with a 7.09-point decrease in the JSPE-HPS score compared to feeling their faculty is empathetic towards patients to a “very great” extent, holding all other variables constant. Feeling that faculty is empathetic toward the patients to a “little extent” was associated with a 12.52-point decrease in the JSPE-HPS score compared to respondents’ that feel their faculty is empathetic towards patients to a “very great extent”, holding all other variables constant. Self-reporting to be “very empathetic” was associated with a 5.71-point increase in JSPE-HPS score, holding all other variables constant. It should be noted that the R2 for this model is 0.16; the adjusted R2 is 0.14. The AIC is 2081.16.

**Table 3:** Multivariable final model

Terms	Coefficient (95% CI);	P-Value
Intercept	116.668 (113.082, 120.253)	< 0.001
Faculty Empathy Toward Patients: To great extent	-2.127 (-5.433, 1.179)	0.206
Faculty Empathy Toward Patients: To some extent	-7.093 (-10.951, -3.235)	< 0.001
Faculty Empathy Toward Patients: To a little extent	-12.518 (-18.664, -6.372)	< 0.001
Self Perception of empathy level: Very Empathetic	5.710 (2.977, 8.443)	< 0.001
Male	-1.155 (-4.275, 1.966)	0.467

Coefficient estimates and p-values resulting from the final linear model.

## Discussion

The primary aim of this study was to assess empathy levels and predictors of empathy among healthcare students at different colleges at a large midwestern public university. Overall empathy levels in our sample measured by the JSPE-HPS score (score = 116) were very similar to a national sample of first year college students of osteopathic medicine (score = 116.5) in the U.S., [33] but slightly above the average obtained by students in one US. allopathic medical school (score = 114).[34] When compared to samples of healthcare professional students in Italy (score = 109) and Australia (score = 110), [35, 36] our results were higher. Our results were all within the regular standard-deviation rate reported for the JSPE-HPS, which is 12 points.[33].

Only few studies have looked at empathy levels among multiple health care providers [36, 37]. Considering the importance of interprofessional education[38] and empathy [39] to achieve better healthcare outcomes, it seems appropriate to assess and analyze empathy levels across different healthcare professions. It makes even more sense to use the same tool for empathy assessment for the different health professions students, and the JSE-HPS has been widely used for these diverse audiences.[33].

In our study, the only factors which were statistically significantly associated with students’ JSPE-HPS scores were students’ self-rated levels of empathy and students’ perception of faculty empathy towards patients.

Not surprisingly, students’ self-rated empathy were also correlated with their JSPE-HPS scores in our sample, as the JSPE-HPS is based on self-reported attitudes. However, research has shown that JSPE-HPS scores may not correlate with patients’ assessments of empathy levels,[40, 41] indicating that patients’ perspectives should be included in the training strategies for empathy development among healthcare students.[40] Strategies include discussing patients’ assessments in debriefing with trainees, using reflection papers to help students understand patients’ frames of reference, and suggesting possible ways to positively respond to patients’ views and opinions. [35]

The association between JSPE-HPS scores and students’ perception of faculty empathy towards patients was consistent with previous studies showing the importance of positive role modeling for cultivating empathy and the bleak effect of negative role modeling and hidden curriculum on the empathy levels of health professional students.[25–27] Students have consistently reported that positive role models of compassion, respect for patients, and altruism have a very profound affirming influence in students’ own conceptions. On the other hand, negative models can add to students’ own disappointment and cynicism.[25] However, students’ recognition of their faculty members’ empathy is very subjective, and it is unclear whether the faculty member(s) who students had in mind was only one person or not. These two points might influence the interpretation of the present results.

Interestingly, the amount of training received (which class students are) and participants’ sex and/or gender were not associated with changes in empathy levels in our sample. In previous studies, empathy has been shown to decrease in health profession schools, [20–22, 42] and females has been shown to present more empathy overall.[32, 36] In part, these results might be due to some limitations of our study. Our study was restricted to one university in the U.S. Midwest region, with a clear predominance of Caucasian females in the sample. Another limitation to be considered is that the survey was taken in the midst of an atypically stressful period during the COVID-19 pandemic, and it is well known that stress levels can influence empathy.[17] Furthermore, the sample size is relatively small (n = 300, 14.4% response rate) and it is fair to expect some sampling bias, as those answering email surveys in a time of increased online activity (due to the pandemic) might already have a more empathetic nature. Therefore, generalizability should be considered with caution.

Nevertheless, the findings provide research-based evidence showing that students’ perception of their faculty’s empathy levels towards students and patients are associated with students’ JSPE-HPS scores when controlling for other variables, reinforcing the importance of faculty role modeling and how the hidden curriculum can play an important role on preparing a compassionate healthcare workforce for the future.

## Conclusion

Overall empathy levels in this study (JSPE-HPS score = 116) was similar to those reported in the literature and within the regular standard-deviation rate reported for the JSPE-HPS. Controlling for other available variables, healthcare students’ view of their faculty’s empathy toward patients and students’ self-reported empathy levels were the only statistically significant variables associated with students’ JSPE-HPS scores.

## Data Availability

The datasets used and/or analysed during the current study are available from the corresponding author on reasonable request.

## List of Abbreviations

JSPE-HPS: Jefferson Scale of Physician Empathy- Health Professions Student version
IRB ID: Institutional Review Board Identification
D3: Third year dental student
BSN: Bachelor of Science in Nursing
DNP: Doctor of Nursing Practice
MSN: Mater’s of Science in Nursing
CNL: Clinical Nurse Leader
RN: Registered Nurse
AIC: Akaike information criterion

## References

[1] Goetz JL, Keltner D, Simon-Thomas E (2010). Compassion: an evolutionary analysis and empirical review. Psychol Bull.

[2] Lamm C, Decety J, Singer T (2011). Meta-analytic evidence for common and distinct neural networks associated with directly experienced pain and empathy for pain. NeuroImage.

[3] Klimecki OM, Leiberg S, Ricard M, Singer T (2014). Differential pattern of functional brain plasticity after compassion and empathy training. Soc Cognit Affect Neurosci.

[4] Lown BA, Rosen J, Martilla J (2011). An agenda for improving compassionate care: a Survey shows about half of patients say such care is missing. Health Aff.

[5] Mercer SW, Jani BD, Maxwell M, Wong SY, Watt GC (2012). Patient enablement requires physician empathy: a cross-sectional study of general practice consultations in areas of high and low socioeconomic deprivation in Scotland. BMC Fam Pract.

[6] Beach MC, Keruly J, Moore RD (2006). Is the quality of the patient-provider relationship associated with better adherence and health outcomes for patients with HIV?. J Gen Intern Med.

[7] Kerse N, Buetow S, Mainous AG, Young G, Coster G, Arroll B (2004). Physician-patient relationship and medication compliance: a primary care investigation. Ann Fam Med.

[8] Pereira L, Figueiredo-Braga M, Carvalho IP (2016). Preoperative anxiety in ambulatory surgery: the impact of an empathic patient-centered approach on psychological and clinical outcomes. Patient Educ Couns.

[9] Dambha-Miller H, Feldman AL, Kinmonth AL, Griffin SJ (2019). Association between Primary Care Practitioner Empathy and Risk of Cardiovascular events and all-cause Mortality among patients with type 2 diabetes: a Population-Based prospective cohort study. Ann Fam Med.

[10] Rakel DP, Hoeft TJ, Barrett BP, Chewning BA, Craig BM, Niu M (2009). Practitioner empathy and the duration of the common cold. Fam Med.

[11] Levinson W, Gorawara-Bhat R, Lamb J (2000). A study of patient clues and physician responses in primary care and surgical settings. JAMA.

[12] Watanabe S, Yoshida T, Kono T, Taketa H, Shiotsu N, Shirai H (2018). Relationship of trainee dentists’ self-reported empathy and communication behaviors with simulated patients’ assessment in medical interviews. PLoS ONE.

[13] Sarinopoulos I, Hesson AM, Gordon C, Lee SA, Wang L, Dwamena F (2013). Patient-centered interviewing is associated with decreased responses to painful stimuli: an initial fMRI study. Patient Educ Couns.

[14] Uziel N, Meyerson J, Giryes R, Eli I. Empathy in dental care - the role of vicarious trauma.Int Dent J. 2019. 10.1111/idj.12487 PMC9379059 31102260

[15] Moore PJ, Adler NE, Robertson PA (2000). Medical malpractice: the effect of doctor-patient relations on medical patient perceptions and malpractice intentions. Western J Med.

[16] Beckman HB, Markakis KM, Suchman AL, Frankel RM (1994). The doctor-patient relationship and malpractice. Lessons from plaintiff depositions. Arch Intern Med.

[17] Thomas MR, Dyrbye LN, Huntington JL, Lawson KL, Novotny PJ, Sloan JA (2007). How do distress and well-being relate to medical student empathy? A multicenter study. J Gen Intern Med.

[18] McClelland LE, Vogus TJ (2014). Compassion practices and HCAHPS: does rewarding and supporting workplace compassion influence patient perceptions?. Health Serv Res.

[19] Singh Ospina N, Phillips KA, Rodriguez-Gutierrez R, Castaneda-Guarderas A, Gionfriddo MR, Branda ME (2019). Eliciting the patient’s agenda- secondary analysis of recorded clinical encounters. J Gen Intern Med.

[20] Neumann M, Edelhauser F, Tauschel D, Fischer MR, Wirtz M, Woopen C (2011). Empathy decline and its reasons: a systematic review of studies with medical students and residents. Acad medicine: J Association Am Med Colleges.

[21] Bellini LM, Baime M, Shea JA (2002). Variation of mood and empathy during internship. JAMA.

[22] Sherman JJ, Cramer A (2005). Measurement of changes in empathy during dental school. J Dent Educ.

[23] Ferri P, Rovesti S, Panzera N, Marcheselli L, Bari A, Di Lorenzo R (2017). Empathic attitudes among nursing students: a preliminary study. Acta bio-medica: Atenei Parmensis.

[24] Carvajal M, Lopez S, Sarabia-Alvarez P, Fontealba J, Padilla M, Sumi J et al. Empathy Levels of Dental Faculty and Students: A Survey Study at an Academic Dental Institution in Chile.J Dent Educ. 2019. 10.21815/JDE.019.124 31235504

[25] Wear D, Zarconi J (2008). Can compassion be taught? Let’s ask our students. J Gen Intern Med.

[26] Burack JH, Irby DM, Carline JD, Root RK, Larson EB (1999). Teaching compassion and respect. Attending physicians’ responses to problematic behaviors. J Gen Intern Med.

[27] Sinclair S, Norris JM, McConnell SJ, Chochinov HM, Hack TF, Hagen NA (2016). Compassion: a scoping review of the healthcare literature. BMC Palliat care.

[28] Rosenzweig J, Blaizot A, Cougot N, Pegon-Machat E, Hamel O, Apelian N (2016). Effect of a person-centered course on the empathic ability of Dental Students. J Dent Educ.

[29] Wündrich M, Schwartz C, Feige B, Lemper D, Nissen C, Voderholzer U (2017). Empathy training in medical students - a randomized controlled trial. Med Teach.

[30] Ozcan CT, Öksüz E, Oflaz F (2018). Improving Empathy in nursing students: a comparative longitudinal study of two curricula. J Korean Acad Nurs.

[31] Williams B, Beovich B (2020). A systematic review of psychometric assessment of the Jefferson Scale of Empathy using the COSMIN risk of Bias checklist. J Eval Clin Pract.

[32] Fields SK, Mahan P, Tillman P, Harris J, Maxwell K, Hojat M (2011). Measuring empathy in healthcare profession students using the Jefferson Scale of Physician Empathy: health provider–student version. J Interprof Care.

[33] Hojat M, DeSantis J, Shannon SC, Mortensen LH, Speicher MR, Bragan L (2018). The Jefferson Scale of Empathy: a nationwide study of measurement properties, underlying components, latent variable structure, and national norms in medical students. Adv Health Sci Educ Theory Pract.

[34] Hojat M, Gonnella JS, Eleven Years of Data on the Jefferson Scale of Empathy-Medical Student Version (JSE-S) (2015). Proxy Norm Data and tentative cutoff scores. Medical principles and practice: international journal of the Kuwait University. Health Sci Centre.

[35] Petrucci C, La Cerra C, Aloisio F, Montanari P, Lancia L (2016). Empathy in health professional students: a comparative cross-sectional study. Nurse Educ Today.

[36] Williams B, Brown T, McKenna L, Boyle MJ, Palermo C, Nestel D (2014). Empathy levels among health professional students: a cross-sectional study at two universities in Australia. Adv Med Educ Pract.

[37] Nunes P, Williams S, Sa B, Stevenson K (2011). A study of empathy decline in students from five health disciplines during their first year of training. Int J Med Educ.

[38] Interprofessional_Education_Collaborative. Core competencies for interprofessional collaborative practice:2016 update 2016 [Available from: https://aamc-meded.global.ssl.fastly.net/production/media/filer_public/70/9f/709fedd7-3c53-492c-b9f0-b13715d11cb6/core_competencies_for_collaborative_practice.pdf.

[39] Trzeciak SM, Compassionomics A (2019). The Revolutionary Scientific evidence that Caring makes a difference.

[40] Bernardo MO, Cecílio-Fernandes D, Costa P, Quince TA, Costa MJ, Carvalho-Filho MA (2018). Physicians’ self-assessed empathy levels do not correlate with patients’ assessments. PLoS ONE.

[41] Bernardo MO, Cecilio-Fernandes D, Lima ARA, Silva JF, Ceccato HD, Costa MJ (2019). Investigating the relation between self-assessment and patients’ assessments of physicians-in-training empathy: a multicentric, observational, cross-sectional study in three teaching hospitals in Brazil. BMJ open.

[42] DeRouen TA, Hujoel P, Leroux B, Mancl L, Sherman J, Hilton T (2008). Preparing practicing dentists to engage in practice-based research. J Am Dent Assoc.

